# Localized activity alternations in periventricular nodular heterotopia‐related epilepsy

**DOI:** 10.1111/cns.14104

**Published:** 2023-02-05

**Authors:** Wenyu Liu, Hesheng Zhang, Xinyu Hu, Dong Zhou, Xintong Wu

**Affiliations:** ^1^ Departments of Neurology, West China Hospital Sichuan University Chengdu China; ^2^ Departments of Radiology, Huaxi MR Research Center (HMRRC), West China Hospital Sichuan University Chengdu China

**Keywords:** activity, epilepsy, pathological mechanism, periventricular nodular heterotopia

## Abstract

**Objective:**

Periventricular nodular heterotopia (PNH) is a common type of heterotopia usually characterized by epilepsy. Previous studies have identified alterations in structural and functional connectivity related to this disorder, but its local functional neural basis has received less attention. The purpose of this study was to combine univariate analysis and a Gaussian process classifier (GPC) to assess local activity and further explore neuropathological mechanisms in PNH‐related epilepsy.

**Methods:**

We used a 3.0‐T scanner to acquire resting‐state data and measure local regional homogeneity (ReHo) alterations in 38 patients with PNH‐related epilepsy and 38 healthy controls (HCs). We first assessed ReHo alterations by comparing the PNH group to the HC group using traditional univariate analysis. Next, we applied a GPC to explore whether ReHo could be used to differentiate PNH patients from healthy patients at an individual level.

**Results:**

Compared to HCs, PNH‐related epilepsy patients exhibited lower ReHo in the left insula extending to the putamen as well as in the subgenual anterior cingulate cortex (sgACC) extending to the orbitofrontal cortex (OFC) [*p* < 0.05, family‐wise error corrected]. Both of these regions were also correlated with epilepsy duration. Furthermore, the ReHo GPC classification yielded a 76.32% accuracy (sensitivity = 71.05% and specificity = 81.58%) with *p* < 0.001 after permutation testing.

**Interpretation:**

Using the resting‐state approach, we identified localized activity alterations in the left insula extending to the putamen and the sgACC extending to the OFC, providing pathophysiological evidence of PNH. These local connectivity patterns may provide a means to differentiate PNH patients from HCs.

## Graphical abstract

PNH‐related epilepsy patients exhibited lower regional homogeneity (ReHo) in the left insula extending to putamen and also the subgenual anterior cingulate cortex (sgACC) extending to orbitofrontal cortex (OFC) [p < 0.05, family‐wise error (FWE) correction]. The epilepsy duration was negatively and independently associated with the ReHo value. Furthermore, the GPC classification regarding ReHo yielded an accuracy of 76.32% (sensitivity = 71.05% and specificity = 81.58%) with p < 0.001 after permutation testing.

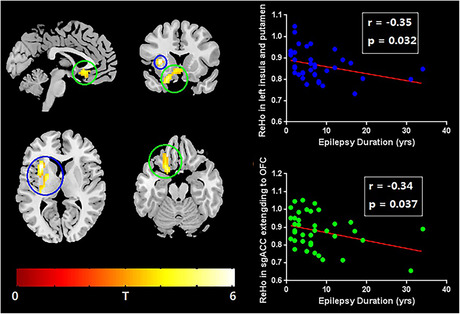

## 1 INTRODUCTION

Cortical developmental is a multistep process that involves proliferation, migration, and organization, each of which can lead to epileptogenic malformations.^1^, ^2^ Periventricular nodular heterotopia (PNH) results from a defective dynamic migration process that leads to ectopic gray matter nodules in abnormal positions along the ventricles.^3^ PNH is a common subtype of malformations of cortical development (MCDs). Bilateral continuous nodules along the ventricles are usually associated with *Filamin A* mutations and are clearly visible, while the majority of sporadic cases with unilateral or single nodules do not have *Filamin A* mutations.^4^

Current clinical PNH diagnoses are mostly based on radiologists' interpretations. Although most PNH can be detected by neuroradiologists using moderate‐to‐high‐quality magnetic resonance imaging (MRI), the process is time‐consuming and requires considerable expertise. Especially in resource‐limited regions, there are still some problems with PNH diagnostic report as the neuroimaging techniques and personal experience of radiologists are key points to diagnose PNH. Machine learning has become increasingly popular over the past decade and has served as a supplementary diagnostic tool for diseases such as glioma^5^ and malignant lung nodule,^6^ as well as PNH and MCDs.^7^ Furthermore, multivariate pattern analysis (MVPA) has been used to develop brain signatures for clinical diagnoses that are more effective than traditional linear models.^8^ A machine learning approach to PNH diagnosis could potentially accelerate and improve conventional neuroradiological interpretation.

The neuroanatomical alterations related to PNH have been well investigated.^9^ However, the functional neural correlates of PNH remain elusive, though previous work demonstrated brain abnormalities in PNH using a seed‐based functional connectivity approach.^10^, ^11^ Local functional analysis could provide an opportunity to discover localized functional disruptions in patient groups without a priori constraints, thus enabling the discovery of previously unconsidered regional brain abnormalities. Regional homogeneity (ReHo) is a voxel‐based physiological metric that reflects local signal similarity and is derived from a blood oxygen level‐dependent (BOLD) functional MRI (fMRI) time course. Recently, ReHo has been shown to be a valid metabolic proxy.^12^ Since brain functional metabolism is significant to both epilepsy diagnosis and epileptogenic foci localization, local functional analyses may further our knowledge of PNH‐related epilepsy's underlying neuropathological mechanisms and may assist in clinical outcome predictions.

Here, we combined both univariate analysis and a Gaussian process classifier (GPC) approach to explore ReHo in PNH. A training stage and a testing stage were both included in our analysis comparing PNH patients and healthy controls (HCs). We hypothesized that the GPC approach to the ReHo maps would (1) be able to discriminate individual patients with PNH from HCs and (2) provide information on neurobiological changes that could help to elucidate the mechanisms that cause PNH.

## 2 METHODS

### 2.1 Participants

Thirty‐eight PNH‐related epilepsy patients were included from neurology department, West China Hospital, Sichuan University. All methods were complying with guidelines and regulations. All subjects provided their informed consent before being enrolled, and this current research was approved by the local ethical committee of West China Hospital, Sichuan University. All of them presented with seizures based on the definition of International League Against Epilepsy.^13^ Thirty‐eight healthy subjects were also enrolled through advertisement in local region. They were age‐ and sex‐matched. The clinical information was collected through standard questionnaire.

### 2.2 MRI data acquisition and preprocessing

3.0‐T MRI scanner (Siemens Trio) was applied to collect fMRI data. A echo‐planar imaging (EPI) sequence was applied to obtain BOLD signal levels of the whole brain. TR: 2000 ms, echo time: 30 ms, flip angle: 90°, slice thickness: 5 mm, no slice gap, field of view: 240 × 240 mm^2^, voxel size: 3.75 × 3.75 × 5 mm^3^, 30 axial slices, 200 volumes, and scan time: 404 s. The included subjects were informed to relax, close eyes, and not to sleep during scanning. Earplugs and foam padding were used to make subjects comfortable.

We adopted the DPABI toolbox^14^ to preprocess the resting‐state fMRI data. Specifically, the first ten time points were excluded in order to confirm the stability of the MRI signals. The remaining 190 volumes were used to perform the slice timing and head motion correction. We used the Friston 24‐parameter model for head motion correction in the current study, because previous publication had reported that the Friston 24‐parameter model is superior to the traditional 6‐parameter model.^15^ The translational and rotational parameters for all of the image data did not exceed ±1.5 mm and ± 1.5 degree, while the mean framewise displacement (FD) value did not exceed 0.2. Afterward, we spatially normalized these image data to the EPI template with the resolution of 3 × 3 × 3 mm^3^. The covariates including the signal from the cerebrospinal fluid, white matter, and global mean signal intensity were regressed out. Finally, we removed the linear trend of the fMRI images and performed the band‐pass filtering (0.01–0.08 Hz).

### 2.3 Regional Homogeneity Calculation

We used the REST software (http://resting‐fmri.sourceforge.net) to conduct the ReHo analysis.^16^ Specifically, we calculated the Kendall coefficient of concordance (KCC) value of the time series between one single voxel with adjacent 26 voxels of its neighbors for creating the ReHo map of each participant. Afterward, a whole‐brain mask was adopted to remove all the non‐brain tissues for standardization. Then, the ReHo of each voxel was further divided by the global mean of ReHo values. Ultimately, all the individual standardized ReHo maps were smoothed by an 8‐mm full‐width half maximum (FWHM) Gaussian kernel.

### 2.4 Univariate Analysis

We used a standard, univariate approach to investigate ReHo alterations between patients with PNH and controls by means of two‐sample *t*‐tests in SPM12 software. The results were thresholded at *p* < 0.001 uncorrected at the voxel level, and a minimum cluster extent of 100 and the statistical threshold of cluster level was set at *p* < 0.05 using family‐wise error (FWE) correction. Brain areas with significant ReHo alterations between two groups were extracted as region of interest for Pearson's correlation analyses with clinical characteristics.

### 2.5 Multivariate Pattern Analysis Approach

We adopted GPC approach to discriminate patients with PNH from controls based on the whole‐brain individual ReHo maps obtained in the previous univariate analysis. The theoretical rationale and implementation details of GPC have been described in previous publications.^17^, ^18^, ^19^ Here, we just provided a brief introduction of this multivariate pattern classification method. The GPC is a probabilistic model on the basis of the Bayesian extension of logistic regression. The main strength of GPC model, compared with other alternative methods such as support vector machines, is that the predicted class is augmented by an estimation of the certainty of the prediction. Based on Bayesian principle, the posterior distribution of functions that represent the training data is identified in an optimal way. This posterior distribution is applied for classifying new examples according to the rules of probability.^20^ Thus, the GPC classification could provide probabilistic class predictions and accurately quantify the predictive confidence assigned to each data point.

In the current study, we used a receiver operating characteristic curve (ROC) for evaluating the classification performance of GPC model. The ROC curve could plot the classifier's true positive rate (sensitivity) against its false‐positive rate (1‐specificity) as the decision threshold is varied. Besides, the leave‐one‐out cross‐validation (LOOCV) strategy^21^ was adopted to assess the stability of the GPC classifier. This LOOCV approach discarded each subject once and used the rest of the participants to train the GPC classifier. Subsequently, the discarded participant pair was used to test the differentiating ability of GPC to reliably distinguish between two groups. Finally, we used a permutation test^22^ to determine the statistical significance of the discrimination accuracy yielded by the GPC classifier. Specifically, the permutation test repeated the discrimination process 1000 times using a different random permutation of the training group labels and counting the number of permutations that achieved the same or higher sensitivity and specificity relative to the true labels. All these MVPA procedures were performed using the PROBID toolbox (http://www.brainmap.co.uk/probid.htm) as implemented in the MATLAB software.

## 3 RESULTS

This study included 38 right‐handed patients with PNH (age (mean ± standard deviation (SD)): 25.45 ± 6.28 years, 16 males, age at seizure onset: 18.1 ± 5.8 years) and 38 age‐ and sex‐matched HCs (age ± SD: 25.50 ± 6.27 years, 16 males). The PNH group had been diagnosed with epilepsy before heterotopic nodules were revealed by MRI. HCs were excluded if MRI scanning showed any structural lesions. We identified no significant differences between the PNH group and the HC group concerning age, sex, handedness, years of education, or mini‐mental state examination (MMSE) performance (*p* > 0.05) (Table 1).

**TABLE 1:** Clinical features of PNH patients and healthy controls.

Clinical features	PNH patients	Healthy controls	*p* value
	(*n* = 38)	(*n* = 38)	
Age (years)	25.68 ± 7.68	25.63 ± 7.39	0.9758
Sex (male/female)	14/24	14/24	1
Education years	10.16 ± 3.13	11.11 ± 2.69	0.1614
Duration (months)	48.58 ± 37.33	‐	‐
Frequency (daily/monthly/yearly)	2/21/15	‐	‐
Seizure type (focal‐only)	30	‐	‐
Medication (mono/duo/poly‐therapy)	11/24/3	‐	‐

Abbreviation: PNH, periventricular nodular heterotopia.

**FIGURE 1:**
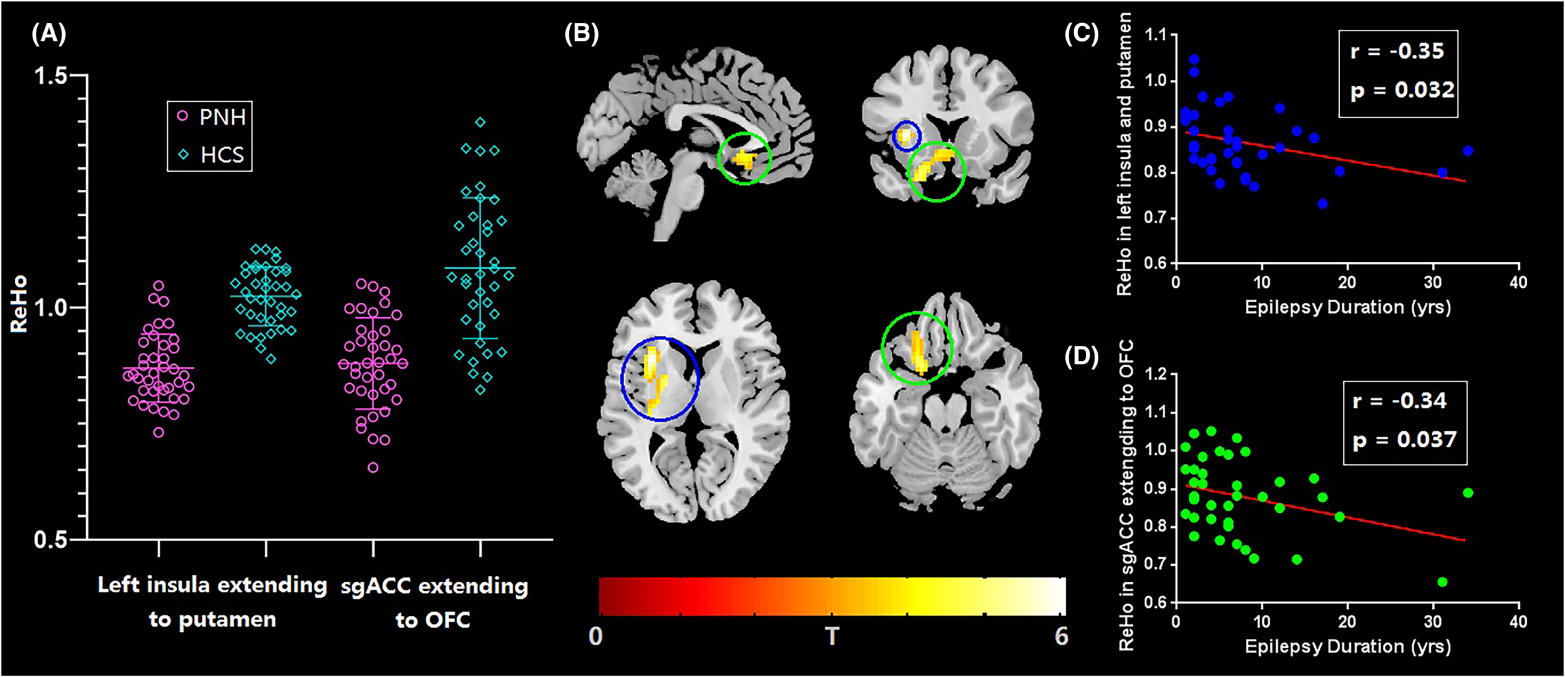
Patients group of PNH showed lower ReHo values in the left insula extending to the left putamen as well as the subgenual anterior cingulate cortex (sgACC) extending to the orbitofrontal cortex (OFC) (*p* < 0.05, FWE correction at the cluster level) (A and B). The epilepsy duration was negatively and independently associated with the ReHo value in the left insula extending to the left putamen (*r* = −0.35, *p* = 0.032) and the ReHo value in the sgACC extending to the OFC (*r* = −0.34, *p* = 0.037) (C and D).

We found that the PNH group had lower ReHo values in the left insula extending to the left putamen as well as in the subgenual anterior cingulate cortex (sgACC) extending to the orbitofrontal cortex (OFC) (*p* < 0.05, FWE correction at the cluster level) (Table 2 and Figure 1A,B). We identified no regions with higher ReHo values. Furthermore, epilepsy duration was negatively and independently associated with the ReHo value in the left insula extending to the left putamen (*r* = −0.35, *p* = 0.032) and the ReHo value in the sgACC extending to the OFC (*r* = −0.34, *p* = 0.037) (Figure 1C,D).

**TABLE 2:** Decreased ReHo in patients with PNH compared with healthy controls.

Region	Side	Voxel size	MNI coordinate			T	*p* *
			x	y	z		
Insula extending to putamen	L	234	−33	15	6	6.05	0.001
			−21	−3	3	5.52	
			−33	−21	−3	5.43	
sgACC extending to OFC	L	184	−21	12	−27	5.25	0.002
			−6	21	−6	4.69	

Abbreviations: MNI, Montreal Neurological Institute; OFC, orbitofrontal cortex; PNH, periventricular nodular heterotopia; ReHo, regional homogeneity; sgACC, subgenual anterior cingulate cortex.

* *p* < 0.05 with whole‐brain family wise error correction at cluster level.

**FIGURE 2:**
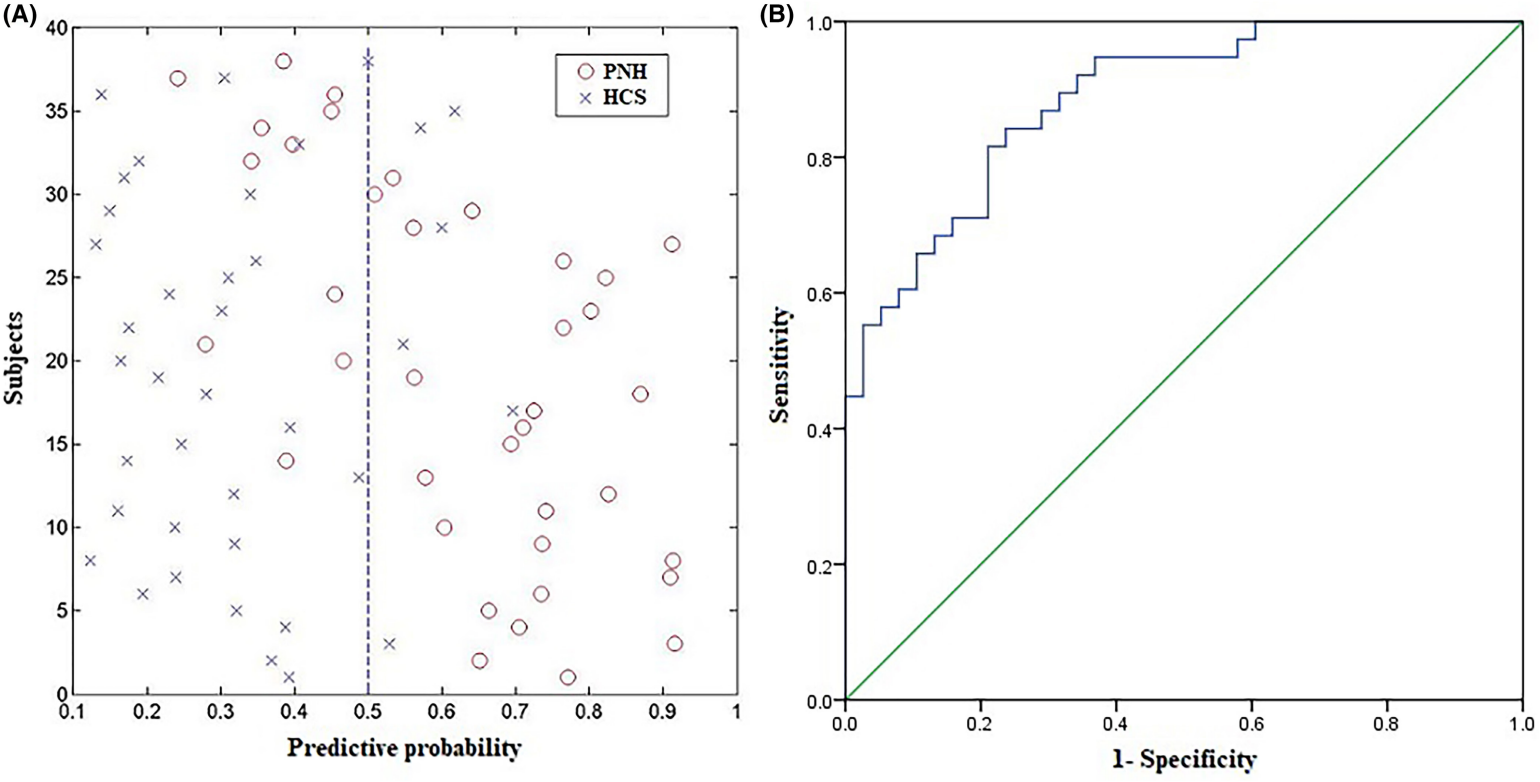
The plotting for Gaussian process classifier (GPC) classification using ReHo maps are presented in left. The receiver operating characteristic (ROC) curve evaluating the GPC classification performance based on ReHo maps are shown in right.

Based on the GPC classification, the ReHo map diagnostic accuracy for distinguishing PNH patients versus HCs was 76.32% (sensitivity = 71.05% and specificity = 81.58%, *p* < 0.001). We constructed GPC classification plots using ReHo maps (Figure 2, left) and a ROC curve evaluating the GPC classification performance based on the ReHo maps (Figure 2, right).

## 4 DISCUSSION

Here, for the first time, we applied both univariate analysis and MVPA to explore ReHo alterations in patients with PNH. We achieved an overall classification accuracy of 76.32% (*p* < 0.001) using the ReHo map‐based GPC classifier and also improved our understanding of the neuropathological mechanisms underlying PNH. These findings may increase diagnostic objectivity and certainty, detecting subtle nodules that may be otherwise overlooked.

Significant progress has been made toward understanding the electrophysiological and functional mechanisms underlying PNH. PNH features ectopic nodules located in the white matter that have been reported to have altered diffusion metrics.^23^ Additionally, functional white matter activity and connectivity have been observed both at rest and during tasks.^24^, ^25^, ^26^, ^27^ However, the local functional neural basis of this disorder is not well understood. In our previous PNH‐related epileptic network investigation, we found that amplitude of low‐frequency fluctuations (ALFFs) had lower amplitude in default mode network (DMN) hubs.^28^ ReHo is another important voxel‐based physiological metric.^29^, ^30^ Recently, voxel‐based BOLD signal metrics at rest have been shown to reflect underlying metabolic demand as measured by positron emission tomography.^12^ This supports the use of fractional ALFF and ReHo as metabolic proxies and demonstrates that localized activity alterations may be related to specific network interactions in PNH‐related epilepsy.

The neuroanatomical alterations and structural connectivity between nodules and the overlying cortex,^31^ as well as internodular synchrony of intracerebral interictal activity, have been extensively explored.^32^ However, we are still lacking comprehensive research underlying PNH‐related epileptic network. In the current study, we found that PNH patients had lower ReHo values in the left insula extending to the left putamen as well as the sgACC extending to the OFC. Furthermore, both regions were correlated with epilepsy duration. Consistent with this, a previous study enrolled Rolandic epilepsy patients and found that the patient group had lower ReHo in the bilateral OFC.^33^ Additionally, altered prefrontal cortex local activity has been widely reported in subtypes such as generalized epilepsy^34^, ^35^ and temporal lobe epilepsy.^36^ A previous task‐based fMRI study revealed that PNH patients had significantly modulated heterotopia as well as widespread cortical activation,^37^ and during a task‐free resting state, unique directionality was observed within an interconnected network.^11^ Another study used seed‐defined nodules that combined structural and functional connectivity and showed disrupted FC in multiple brain areas including the cerebellum,^10^ while an additional study used seeds defined by altered ALFF areas and showed higher FC in the fronto‐parieto‐limbic neurocircuitry and lower FC in the DMN.^28^

Methodological differences are one possible reason for the inconsistencies among PNH studies. We evaluated functional changes in PNH by assessing local functional disruptions in PNH‐related epilepsy groups without a priori constraints rather than focusing on nodule dysregulation as in seed‐based functional connectivity studies. ROI definitions for seed‐based analysis also differed. Our previous study^28^ defined areas with significant ALFF alterations that were then used as seeds in whole‐brain FC analyses, while Christodoulou et al. identified heterotopic nodules as seed ROIs for functional connectivity analysis.^10^ Compared to analysis in particular tasks,^38^ resting‐state fMRI is relatively easy to obtain and may have different, potentially even broader, significance. Additionally, the current study included a larger number of PNH patients compared to previous studies.

Though PNH diagnostic report mostly based on neuroimaging technique and radiologists, the intensity of field, slice thickness, nodule size, and position all influence the detection of the PNH. PNH may be also coexisted with other cortical or extracortical neurodevelopmental deformities.^39^ In these conditions, PNH were more likely to be neglected compared to other diffuse malformation. Classification analyses comparing PNH patients and HCs could improve diagnostic accuracy, which is of clinical significance. In a recent study,^7^ the authors developed and tested a deep learning model to automatically detect MCD and further distinguish between diffuse cortical malformation, PNH, and normal MRI at a clinically useful performance level. Neuroimaging is promising to impact clinical practice and public health, with the potential to transform the role of neuroimaging in clinical applications. Computer‐assisted detection of PNH could potentially accelerate and improve conventional neuroradiological interpretation.

Our study has several limitations. First, the patient sample size was relatively small. Although PNH is a rare disorder, we only enrolled adult PNH patients with separate and small‐volume nodules. This made study inclusion more difficult. Second, treatment effects on our findings cannot be ruled out, since the majority of PNH patients were on epilepsy medication. Third, it is unclear whether the ReHo map GPC classification would distinguish PNH from other cortical development malformation subtypes. Finally, we could not identify an association between ReHo anomalies and cognitive deficits because we did not perform detailed neuropsychological tests for PNH patients to evaluate cognitive function.

## AUTHOR CONTRIBUTIONS

Conception and design: Wenyu Liu and Xintong Wu; Administrative support: Xintong Wu and Dong Zhou; Provision of study materials or patients: Xintong Wu; Collection and assembly of data: Hesheng Zhang; Data analysis and interpretation: Xintong Wu and Xinyu Hu; Manuscript writing: Wenyu Liu; Final approval of manuscript: All authors.

## CONFLICT OF INTEREST STATEMENT

All authors have completed the ICMJE uniform disclosure form. The authors all have no conflicts of interest to declare.

## Data Availability

The data that support the findings are available upon reasonable request.
